# Reverse Correlation Uncovers More Complete Tinnitus Spectra

**DOI:** 10.1109/OJEMB.2023.3275051

**Published:** 2023-05-18

**Authors:** Alec Hoyland, Nelson V. Barnett, Benjamin W. Roop, Danae Alexandrou, Myah Caplan, Jacob Mills, Benjamin Parrell, Divya A. Chari, Adam C. Lammert

**Affiliations:** Department of Biomedical Engineering (BME)Worcester Polytechnic Institute (WPI) 8718 Worcester MA 01609 USA; Clarifai, Inc. Wilmington DE 19808 USA; BMEWorcester Polytechnic Institute 8718 Worcester MA 01609 USA; Neuroscience ProgramWorcester Polytechnic Institute 8718 Worcester MA 01609 USA; Stritch School of MedicineLoyola University Chicago 2456 Chicago IL 60660 USA; Department of Communication Sciences and Disorders and the Waisman CenterUniversity of Wisconsin 5228 Madison WI 53707 USA; University of Massachusetts Chan Medical School 12262 Worcester MA 01609 USA; Massachusetts Eye and Ear Infirmary 1866 Boston MA 02114 USA

**Keywords:** Reverse correlation, tinnitus, behavioral assay

## Abstract

*Goal:* This study validates an approach to characterizing the sounds experienced by tinnitus patients via reverse correlation, with potential for characterizing a wider range of sounds than currently possible. *Methods:* Ten normal-hearing subjects assessed the subjective similarity of random auditory stimuli and target tinnitus-like sounds (“buzzing” and “roaring”). Reconstructions of the targets were obtained by regressing subject responses on the stimuli, and were compared for accuracy to the frequency spectra of the targets using Pearson's $r$. *Results:* Reconstruction accuracy was significantly higher than chance across subjects: buzzing: $0.52 \pm 0.27$ (mean $\pm$ s.d.), $t(9) = 5.766$, $p < 0.001$; roaring: $0.62 \pm 0.23$, $t(9) = 5.76$, $p < 0.001$; combined: $0.57 \pm 0.25$, $t(19) = 7.542$, $p < 0.001$. *Conclusion:* Reverse correlation can accurately reconstruct non-tonal tinnitus-like sounds in normal-hearing subjects, indicating its potential for characterizing the sounds experienced by patients with non-tonal tinnitus.

## I. Introduction

Tinnitus the perception of sound in the absence of any corresponding external stimulus—affects up to 50 million people in the U.S. [1], a third of whom experience functional cognitive impairment and diminished quality of life [2], [3]. Clinical guidelines for tinnitus management involve targeted exposure to external sounds as part of *sound therapy* or *cognitive behavioral therapy* [4]. Critically, patient outcomes improve when the employed external sounds are closely informed by the patient's internal tinnitus experience [5], [6], [7], [8]. However, existing strategies for characterizing tinnitus sounds, such as *pitch matching* (PM), are best suited for patients whose tinnitus resembles pure tones (e.g., ringing) [9], [10], [11], [12]. There is a need for methods to characterize tinnitus sounds in the estimated 20–50% of patients with nontonal (e.g., buzzing, roaring) tinnitus [13], [14].

Nontonal tinnitus sounds are presumed to be complex and heterogeneous [12], although few characteristics have been firmly established. Therefore, we base our approach on *reverse correlation* (RC), an established behavioral method [15], [16], [17] for estimating internal perceptual representations that is unconstrained by prior knowledge about the representations themselves. RC asks participants to render subjective judgments over ambiguous stimuli, and reconstructs the latent representation by regressing subject responses onto the stimuli. RC is closely related to Wiener theory, which has inspired “white-noise” approaches to system characterization in physiology [18], [19] and engineering [20].

Here, we validate RC as a method for characterizing individuals' internal representations of tinnitus more completely. To that end, normal-hearing participants completed an augmented RC experiment, comparing random stimuli to a target tinnitus-like sound, yielding frequency spectrum estimates of their tinnitus representation. The estimated spectra were subsequently validated against the target tinnitus sounds. Our results demonstrate, for the first time, that tinnitus-like sounds with complex spectra can be accurately estimated using RC.

## II. Materials and Methods

### A. Stimuli

The frequency space of the stimuli was partitioned into $b=8$ Mel-spaced frequency bins, which divide the frequency space between $[100,\;13,000]$ Hz into contiguous segments of equal amplitude (i.e., “rectangular” bins). Reconstruction detail increases with $b$, but $b=8$ provides a good approximation to the chosen target sounds (*cf*. Fig. 3).

**Fig. 1.**
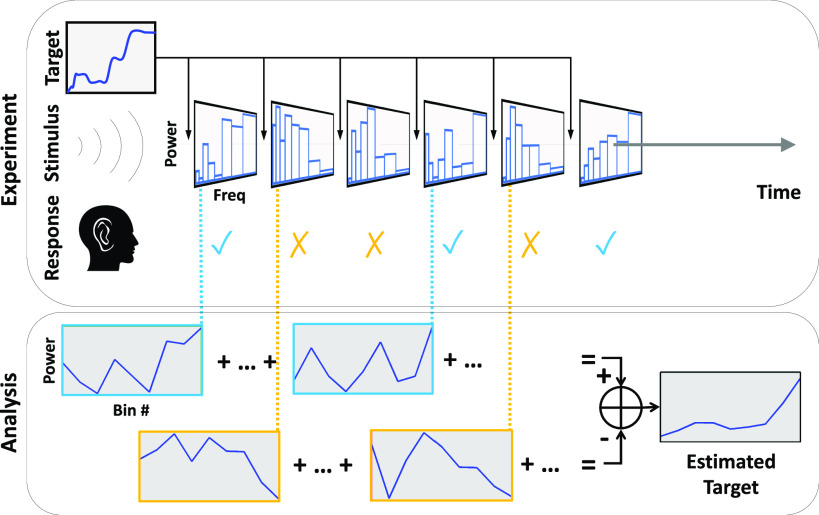
Diagram of the experimental protocol. Subjects listen to a series of random stimuli, each preceded by a target sound. Subjects compare the stimulus to the target, and respond either “yes” or “no” depending on their perceived similarity. The recorded stimulus-response pairs are used to form an estimate of the target.

**Fig. 2.**
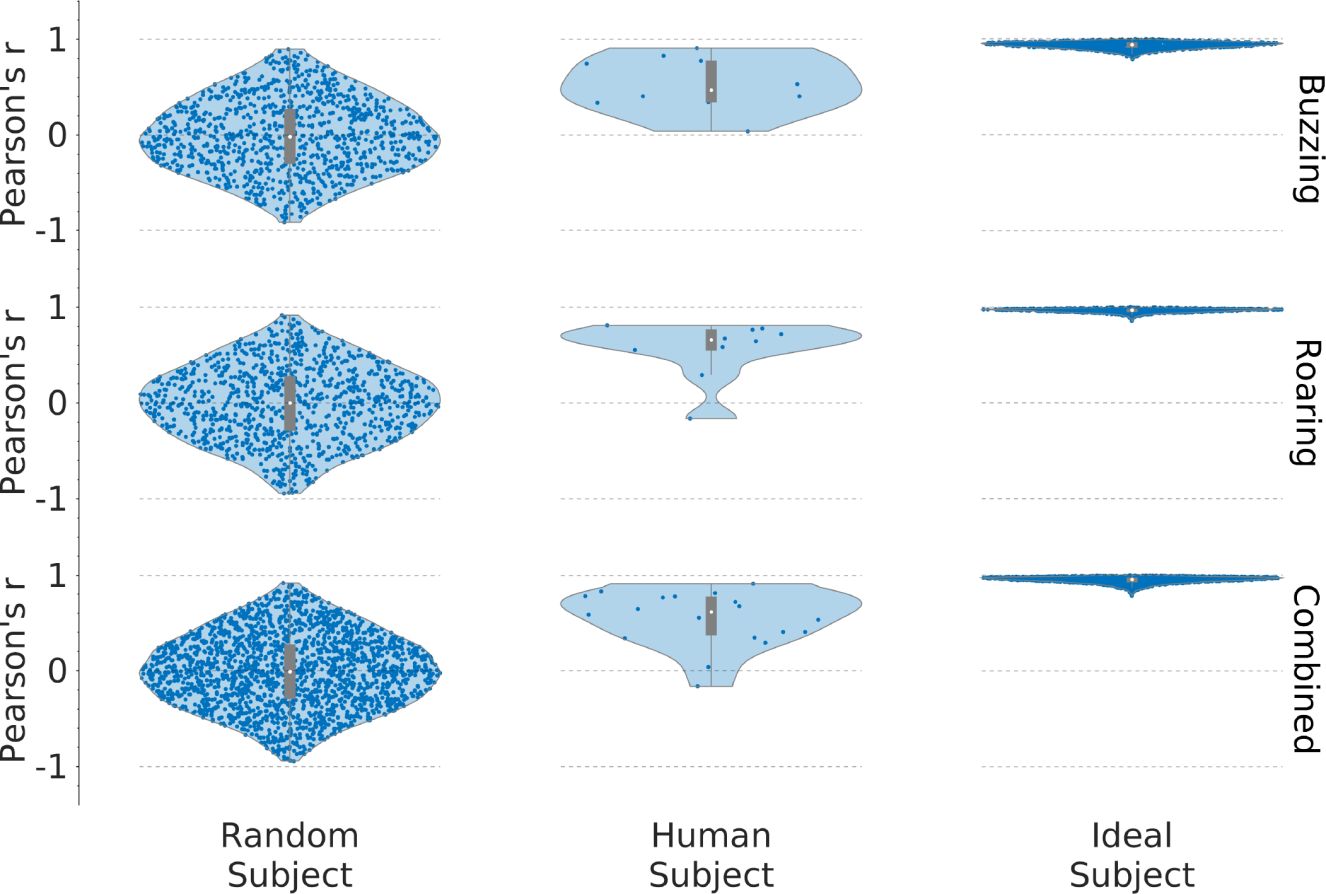
Human reconstruction accuracy is significantly above baseline, but is not optimal. Random, human, and ideal reconstruction accuracies are shown as violin plots with box plots overlaid. The median is a white dot, the ordinate of the blue points are the Pearson's $r$ values.

**Fig. 3.**
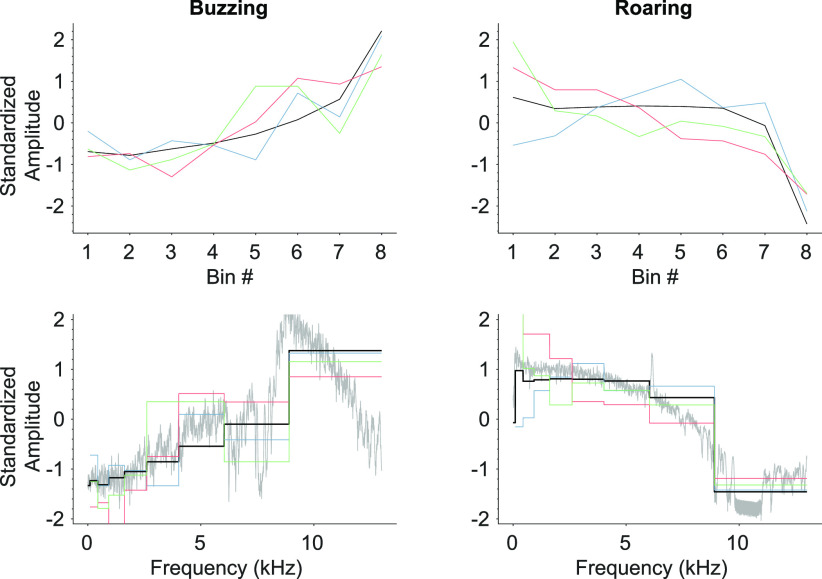
Reconstructions of the tinnitus spectra capture many salient features of the target sounds. The black trace indicates the target, while colored traces plot exemplar human reconstructions. The top row shows standardized power levels within each frequency bin. The bottom row maps the 8-dimensional bin domain to a 11025-dimensional frequency domain with the unbinned power spectral density of the targets shown in gray.

For each stimulus, [2,7] bins were randomly “filled” with power 0 dB. “Unfilled” bins were assigned $-100$ dB. All frequencies were assigned random phase. Inverse Fourier transform of the constructed spectrum yields a 500 ms stimulus waveform.^1^^1^Software for the experiments and analysis was written in MATLAB and is freely available at https://github.com/alec-hoyland/tinnitus-reconstruction/

### B. Target Sounds

Two spectrally complex and complementary target sounds (“buzzing” and “roaring”) were downloaded from the American Tinnitus Association [21] and truncated to 500 ms in duration (power-spectral densities are displayed in the botton subplots of Fig. 3).

### C. Experiment

Ten ($n=10$) normal-hearing subjects listened to *A-X* trials containing a target sound ($A$) followed by a stimulus ($X$). $X$ was randomly generated for each trial, while $A$ remained the same within a block of 100 trials (Fig. 1). Subjects completed two (2) blocks per target sound ($p=200$ total trials per subject). Subjects were told that some stimuli had $A$ embedded in them, and were instructed to respond “yes” to such stimuli, otherwise “no.” Subjects listened over earphones at a self-determined comfortable level. Presentation level was not recorded in this study. Procedures were approved by the UMass IRB.

### D. Reconstruction

A subject performing $p$ RC trials with $b$ frequency bins produces a stimulus matrix $\Psi \in \mathbb {R}^{p \times b}$ and a response vector $y \in \lbrace 1,-1\rbrace ^{p}$, where 1 corresponds to a “yes” response and $-1$ to a “no.” RC classically assumes the subject response model:

\begin{equation*}
y = sign(\Psi x), \tag{1}
\end{equation*}

where $x \in \mathbb {R}^{\mathrm{b}}$ is the subject's internal representation of interest (i.e., of their tinnitus). Inverting this model yields:

\begin{equation*}
\hat{x} = \frac{1}{n} \Psi ^{\mathrm{T}} y \tag{2}
\end{equation*}

which is a restricted form of the Normal equation under the assumption that the stimulus dimensions are uncorrelated [16].

### E. Validation

The experimental paradigm allows for direct validation of the reconstructions $\hat{x}_{buzzing}$ and $\hat{x}_{roaring}$. We represent the spectra of the target sounds as vectors $s_{buzzing} \in \mathbb {R}^{\mathrm{b}}$ and $s_{roaring} \in \mathbb {R}^{\mathrm{b}}$ using the same frequency bins as the stimulus with power equal to the mean power at frequencies within that bin. Pearson's $r$ between $s_{buzzing}$ and $s_{roaring}$ and their corresponding reconstructions quantifies reconstruction accuracy. One-sample t-tests were performed on the mean Fisher-transformed Pearson's $r$ values across subjects to assess significant differences from zero.

### F. Synthetic Subjects

To establish bounds on human performance, additional experiments were run with two simulated subjects who give either *ideal* or *random* responses. Each experiment ran for $p=200$ trials and was repeated 1000 times.

The *ideal* subject gives responses following:

\begin{equation*}
y_{i} = {\begin{cases}1 & \text{if } \Psi _{i} s \geq Q(0.5; \Psi s) \\
 -1 & \text{otherwise} \end{cases}} \tag{3}
\end{equation*}

for $i \in 1,{\ldots }, p$, where $Q(x, y)$ is the quantile function for $x \in [0, 1]$ of the similarity calculation $\Psi s$, and $\Psi _{i}$ is the $i\text{th}$ column of $\Psi$. Thus, the ideal subject has precise knowledge of every stimulus and responds according to (3). The *random* subject responds $y_{i} \in \lbrace 1,-1\rbrace$ with uniform random probability, thus ignoring the stimulus entirely.

## III. Results

Fig. 2 shows the distribution of Pearson's $r$ for human, ideal, and random subject responses. Human accuracy is statistically significantly higher than random chance and for some subjects, approaches the ideal case. Accuracy from the random subject was $0.00 \pm 0.44$ (mean $\pm$ st.dev.) for buzzing and $0.00 \pm 0.39$ for roaring, while mean accuracy from human responses was significantly different from 0 in all conditions: buzzing: $0.52 \pm 0.27$, $t(9) = 5.766$, $p < 0.001$; roaring: $0.62 \pm 0.23$, $t(9) = 5.76$, $p < 0.001$; combined: $0.57 \pm 0.25$, $t(19) = 7.542$, $p < 0.001$. From Fig. 2, it appears that the distribution of buzzing results differs from that of the roaring results, however the difference between buzzing and roaring is not statistically significant (two-way ANOVA across subjects ($F(13) = 2.94$, $p > 0.05$) and target signals ($F(1) = 2.44$, $p > 0.05$)). Fig. 3 plots the most accurate human reconstructions over the target sound spectra.

## IV. Conclusion

Our results show that RC can accurately reconstruct the frequency spectrum of tinnitus-like sounds relevant to non-tonal tinnitus, and therefore represent a proof of concept for using RC to characterize non-tonal tinnitus. Subjects completed the required number of trials within ten minutes, indicating that this procedure could be conducted within a single clinical visit. RC may therefore be useful as the basis for a clinical assay to characterize a wider variety of tinnitus percepts than currently possible. Reconstruction accuracies observed here are below the simulated ideal, which may be attributed to noisy responses universally observed in applications of RC, and which may be mitigated by further optimizing the experimental protocol, stimulus generation, and reconstruction method. For example, recent approaches to improving RC reconstruction methods can boost efficiency, noise robustness and overall accuracy [22]. Future work will focus on more comprehensive validation of this approach, using larger sample sizes, more target sounds, and stricter control of sound presentation level.

## References

[1] J. M. Bhatt, H. W. Lin, and N. Bhattacharyya, “Prevalence, severity, exposures, and treatment patterns of tinnitus in the United States,” JAMA Otolaryngol.–Head Neck Surg., vol. 142, no. 10, pp. 959–965, Oct. 2016. 27441392 10.1001/jamaoto.2016.1700 PMC5812683

[2] D. M. Nondahl , “The impact of tinnitus on quality of life in older adults,” J. Amer. Acad. Audiol., vol. 18, no. 3, pp. 257–266, Mar. 2007. 17479618 10.3766/jaaa.18.3.7

[3] S. Tegg-Quinn, R. J. Bennett, R. H. Eikelboom, and D. M. Baguley, “The impact of tinnitus upon cognition in adults: A systematic review,” Int. J. Audiol., vol. 55, no. 10, pp. 533–540, Oct. 2016. 27240696 10.1080/14992027.2016.1185168

[4] D. E. Tunkel , “Clinical practice guideline: Tinnitus,” Otolaryngol.–Head Neck Surg., vol. 151, no. 2_suppl, pp. S1–S40, Oct. 2014. 25273878 10.1177/0194599814545325

[5] H. Okamoto, H. Stracke, W. Stoll, and C. Pantev, “Listening to tailor-made notched music reduces tinnitus loudness and tinnitus-related auditory cortex activity,” Proc. Nat. Acad. Sci., vol. 107, no. 3, pp. 1207–1210, Jan. 2010. 20080545 10.1073/pnas.0911268107 PMC2824261

[6] R. Schaette, O. König, D. Hornig, M. Gross, and R. Kempter, “Acoustic stimulation treatments against tinnitus could be most effective when tinnitus pitch is within the stimulated frequency range,” Hear. Res., vol. 269, no. 1, pp. 95–101, Oct. 2010. 20619332 10.1016/j.heares.2010.06.022

[7] A. Stein , “Inhibition-induced plasticity in tinnitus patients after repetitive exposure to tailor-made notched music,” Clin. Neuriophysiol., vol. 126, no. 5, pp. 1007–1015, May 2015. 10.1016/j.clinph.2014.08.017 25441152

[8] S. Schoisswohl, J. Arnds, M. Schecklmann, B. Langguth, W. Schlee, and P. Neff, “Amplitude modulated noise for tinnitus suppression in tonal and noise-like tinnitus,” Audiol. Neurotol., vol. 24, no. 6, pp. 309–321, 2019. 10.1159/000504593 PMC6959056 31905364

[9] P. Neff, B. Langguth, M. Schecklmann, R. Hannemann, and W. Schlee, “Comparing three established methods for tinnitus pitch matching with respect to reliability, matching duration, and subjective satisfaction,” Trends Hear., vol. 23, 2019, Art. no. 2331216519887247. 10.1177/2331216519887247 PMC6900670 31805822

[10] J. A. Henry, K. E. James, K. Owens, T. Zaugg, E. Porsov, and G. Silaski, “Auditory test result characteristics of subjects with and without tinnitus,” J. Rehabil. Res. Develop., vol. 46, no. 5, pp. 619–632, 2009. 10.1682/jrrd.2008.11.0157 19882495

[11] O. C. Ukaegbe, F. T. Orji, B. C. Ezeanolue, J. O. Akpeh, and I. A. Okorafor, “Tinnitus and its effect on the quality of life of sufferers: A Nigerian cohort study,” Otolaryngol.–Head Neck Surg., vol. 157, no. 4, pp. 690–695, 2017. 28695761 10.1177/0194599817715257

[12] D. Vajsakovic, M. Maslin, and G. D. Searchfield, “Principles and methods for psychoacoustic evaluation of tinnitus,” in The Behavioral Neuroscience of Tinnitus. Berlin, Germany: Springer, Feb. 2021, pp. 419–451. 10.1007/7854_2020_211 33550568

[13] J. A. Henry, “Measurement” of tinnitus,” Otol. Neurotol., vol. 37, no. 8, Sep. 2016, Art. no. e276. 10.1097/MAO.0000000000001070 27518136

[14] A. Noreña, C. Micheyl, S. Chéry-Croze, and L. Collet, “Psychoacoustic characterization of the tinnitus spectrum: Implications for the underlying mechanisms of tinnitus,” Audiol. Neuro- otol., vol. 7, pp. 358–69, Nov. 2002. 10.1159/000066156 12401967

[15] A. Ahumada and J. Lovell, “Stimulus features in signal detection,” J. Acoustical Soc. Amer., vol. 49, no. 6B, pp. 1751–1756, Jun. 1971.

[16] F. Gosselin and P. G. Schyns, “Superstitious perceptions reveal properties of internal representations,” Psychol. Sci., vol. 14, no. 5, pp. 505–509, Sep. 2003. 12930484 10.1111/1467-9280.03452

[17] W. O. Brimijoin, M. A. Akeroyd, E. Tilbury, and B. Porr, “The internal representation of vowel spectra investigated using behavioral response-triggered averaging,” J. Acoustical Soc. Amer., vol. 133, no. 2, pp. EL118–EL122, Feb. 2013. 10.1121/1.4778264 PMC3864535 23363191

[18] D. Ringach and R. Shapley, “Reverse correlation in neurophysiology,” Cogn. Sci., vol. 28, no. 2, pp. 147–166, 2004.

[19] P. Z. Marmarelis and V. Z. Marmarelis, “The white-noise method in system identification,” in Analysis of Physiological Systems: The White-Noise Approach (Computers in Biology and Medicine Series), P. Z. Marmarelis and V. Z. Marmarelis, Eds. Boston, MA, USA: Springer, 1978, pp. 131–180.

[20] L. Ljung, System Identification: Theory for the User. London, U.K.: Pearson Education, Dec. 1998.

[21] “Listen to sample tinnitus sounds (ATA),” Jun. 14, 2022. [Online]. Available: https://www.ata.org/listen-sample-tinnitus-sounds

[22] A. Compton, B. W. Roop, B. Parrell, and A. C. Lammert, “Stimulus whitening improves the efficiency of reverse correlation,” Behav. Res. Methods, to be published, doi: 10.3758/s13428-022-01946-w. PMC10556169 36038814

